# Performance of a closed-loop glucose control system, comprising a continuous glucose monitoring system and an AI-based controller in swine during severe hypo- and hyperglycemic provocations

**DOI:** 10.1007/s10877-020-00474-2

**Published:** 2020-01-31

**Authors:** Jeremy DeJournett, Michael Nekludov, Leon DeJournett, Mats Wallin

**Affiliations:** 1 Ideal Medical Technologies, 18 N Kensington Rd, Asheville, NC 28804 USA; 2 Karolinska University Hospital, Karolinska Universitetssjukhuset, Eugeniavägen 3, 171 76 Solna, Sweden; 3 Maquet Critical Care AB, Röntgenvägen 2, 17154 Solna, Sweden

**Keywords:** Artificial intelligence, Continuous glucose monitor, Closed loop glucose control system artificial pancreas, Hypoglycemia, Intensive care unit

## Abstract

Intensive care unit (ICU) patients develop stress induced insulin resistance causing hyperglycemia, large glucose variability and hypoglycemia. These glucose metrics have all been associated with increased rates of morbidity and mortality. The only way to achieve safe glucose control at a lower glucose range (e.g., 4.4–6.6 mmol/L) will be through use of an autonomous closed loop glucose control system (artificial pancreas). Our goal with the present study was to assess the safety and performance of an artificial pancreas system, composed of the EIRUS (Maquet Critical Care AB) continuous glucose monitor (CGM) and novel artificial intelligence-based glucose control software, in a swine model using unannounced hypo- and hyperglycemia challenges. Fourteen piglets (6 control, 8 treated) underwent sequential unannounced hypoglycemic and hyperglycemic challenges with 3 IU of NovoRapid and a glucose infusion at 17 mg/kg/min over the course of 5 h. In the Control animals an experienced ICU physician used every 30-min blood glucose values to maintain control to a range of 4.4–9 mmol/L. In the Treated group the artificial pancreas system attempted to maintain blood glucose control to a range of 4.4–6.6 mmol/L. Five of six Control animals and none of eight Treated animals experienced severe hypoglycemia (< 2.22 mmol/L). The area under the curve 3.5 mmol/L was 28.9 (21.1–54.2) for Control and 4.8 (3.1–5.2) for the Treated animals. The total percent time within tight glucose control range, 4.4–6.6 mmol/L, was 32.8% (32.4–47.1) for Controls and 55.4% (52.9–59.4) for Treated (p < 0.034). Data are median and quartiles. The artificial pancreas system abolished severe hypoglycemia and outperformed the experienced ICU physician in avoiding clinically significant hypoglycemic excursions.

## Introduction

The importance of tight glucose control (TGC) in ICU patients first came to light in 2001 with Van den Berge’s large prospective study that demonstrated improved morbidity and mortality rates when ICU patients had their blood glucose levels controlled to a normal range through use of an intravenous insulin infusion [1]. A subsequent study performed by Krinsley in 2004 [2] confirmed these findings, as have other follow-on studies performed in surgical, medical, trauma, and coronary care ICU settings [3–6]. However, the initial TGC enthusiasm was tempered by subsequent reports from large follow-up studies with less favorable outcomes [7, 8]. These studies revealed that applying TGC in common ICUs, where blood glucose measuring methods with poor precision and accuracy commonly are used, caused a higher incidence of deleterious severe hypoglycemic (< 2.2 mmol/L) events. As both moderate and severe hypoglycemia have been associated with increasing ICU mortality rates, avoiding these episodes must be a priority of any glucose control method [9–11].

The original approach to glucose control was paper-based intravenous insulin dosing protocols [12]. Subsequent methods involved use of software/web-based insulin dosing calculators [13–16]. These are all open loop methods that require manual: (1) measurement of the glucose level, (2) input of this level into the insulin dosing calculator, and (3) adjustment of the intravenous infusion rate.

While some software-based insulin dosing calculators represent an improvement over paper-based protocols with regards to severe hypoglycemia and time in range [17, 18], most are still are not capable of eliminating the risk for long-lasting and deep hypoglycemia, minimizing the time spent in moderate hypoglycemia (2.22–3.89 mmol/L), while simultaneously improving upon other glucose metrics associated with ICU mortality rates such as time spent in control range 3.89–7.78 mmol/L and glucose variability [19, 20]. Only a closed-loop glucose control system based on an accurate measuring device can abolish the risk for severe hypoglycemia and minimize time spent in moderate hypoglycemia, while simultaneously maximizing time spent in a desired control range and minimizing glucose variability. Such a system consists of three main subsystems: (1) a reliable continuous glucose monitor with good precision and short lag time, (2) glucose control software, and (3) intravenous infusion pumps.

The EIRUS system (Maquet Critical Care AB) is a CGM system designed for use during surgery and in the ICU. This system is based on a triple lumen central venous catheter with two extra thinner lumens whose orifices are placed underneath a microdialysis membrane glued just above the infusion lumens, combined with an ex-vivo biosensor for glucose and lactate, and a monitor. It has undergone clinical validation, has proven good accuracy and precision, and has been available on the European market for some years [21–23]. Ideal Medical Technologies (IMT) has developed a glucose control algorithm based on artificial intelligence (AI) for use in the ICU setting. This AI-based glucose control software was patterned after the native glucose control system [24], and it has already been successfully tested in a simulation environment [25, 26]. In addition, it was previously integrated with the Dexcom G4 sensor (non-CE-marked for use in the ICU) and two syringe pumps to create a closed loop glucose control system (artificial pancreas). This prototype system was tested in a small pilot study of severe stress induced hyperglycemia in a swine model, and was shown to be both safe and effective [27]. Currently used infusion pumps are accurate and reliable enough to complete a closed-loop glucose control system.

The objective of the present swine study was to investigate the safety and performance of an autonomous closed-loop glucose control system, comprised of the EIRUS CGM and an AI-based glucose controller, and compare it with an ICU physician using a clinical conventional protocol, when the animals received an overdose of insulin and a subsequent unphysiological bolus of 20% Dextrose.

## Materials and methods

The study was performed at the Hedenstierna Laboratory, Uppsala University, Uppsala, Sweden, after approval from the Uppsala Animal Ethics Committee Sweden (case number 5.8.18-11795/2018). The study was carried out in accordance with EU Directive 2010/63/EU for animal experiments. Seventeen domestic-breed piglets of both sexes, median weight 30.0 kg (range 28.0–32.1 kg), were included and handled according to the animal experimentation guidelines of the Uppsala Animal Ethics Committee. The three first animals were used to investigate and tailor the insulin and glucose doses needed to create hypoglycemia (< 3.5 mmol/L) and hyperglycemia (> 10 mmol/L) during the experiment, and they were not included in the statistical analysis. The subsequent 14 pigs that completed the study were divided into two groups, 6 controls and 8 in the treated (AI controller) group. The pigs were sedated with atropine (NM Pharma AB, Sweden) 0.04 mg/kg, tiletamine-zolazepam (Zoletil, Vibrac Laboratories, Carros, France) 6 mg/kg, and xylazine chloride (Rompun, Bayer AG, Germany) 2.2 mg/kg administered IM. Anesthesia was then maintained with ketamine (30 mg/kg/h), midazolam (0.1 mg/kg/h), and fentanyl (4 mcg/kg/h). Rocuronium (2 mg/kg/h) was used for muscle relaxation. Ringer’s acetate (10 ml/kg/h) was administered throughout the experiment. All pigs were tracheostomized and ventilated in pressure-regulated volume control mode with a tidal volume of 10 ml/kg, PEEP 5 cm H_2_O and FiO_2_ 40%. An EIRUS micro-dialysis central venous catheter (CVC) was inserted via the right jugular vein to the superior caval vein. A second EIRUS micro-dialysis CVC was inserted via the right femoral vein to reveal potential interferences between the measurement and the glucose infusion and as a back-up system in case of technical problems with the primary sampling line in the caval vein. The right carotid artery was cannulated for pressure recordings and blood sampling. A urinary Foley catheter was inserted into the bladder.

**Fig. 1:**
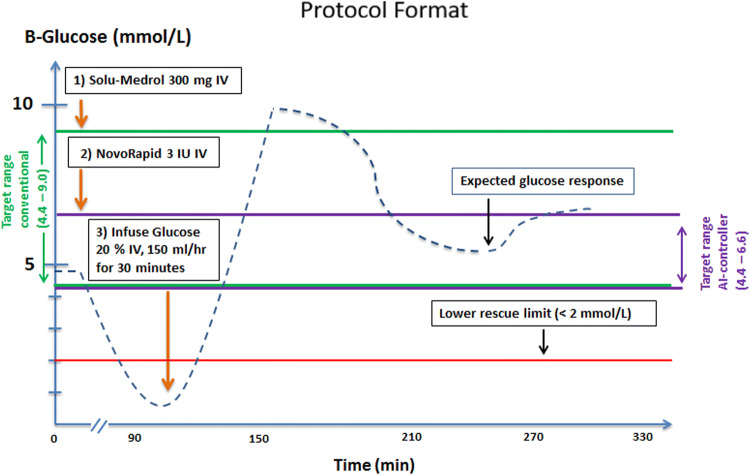
Protocol format. Dashed line represents expected glucose response to study interventions. Severe hypoglycemia was expected at point of time 100 min based on insulin injection (3 units) given at point 20 min. A hyperglycemic excursion greater than 10 mmol/L was induced by the glucose infusion of 17 mg/kg/min given between point 110 and 140 min with an expected maximum at point 150 min. A rebound hypoglycemia was anticipated about point 240 min based on the insulin therapy used to treat the hyperglycemic excursion. The formal study starts at point of time 0 min. Total study duration was 300 min. Observe that the target range for the AI-controller (4.4–6.6 mmol/L) is different and narrower than the target range for the conventional treatment (4.4–9.0)

The protocol is presented in Fig. 1.

To mimic the insulin resistance seen in ICU patients, all animals received a high dose, 300 milligrams, of Solu-Medrol (Pfizer AB) intravenously (IV). Hypoglycemia (< 3.5 mmol/L) was induced by an IV injection of 3 IU NovoRapid (Novo Nordisk) given at the same time as Solu-Medrol—both were given at time 20 min. Hyperglycemia (> 10 mmol/L) was created by an infusion of D20, 75 mL administered over 30 min (time 110–140 min). This represents 15 grams of glucose, which given the average animal weight of 29.1 kg is equivalent to a glucose infusion of 17.2 mg/kg/min for a period of 30 min. The reason for inducing hyperglycemia was to assess the AI controller’s ability to avoid “rebound” hypoglycemia as a result of its attempt to treat the hyperglycemic state. The amount of glucose infused to produce hyperglycemia was beyond the maximum cellular uptake rate of glucose (at least in man), thus hyperglycemia was intentionally unavoidable [28]. Both the 3 IU of insulin and 15 g infusion of glucose were unannounced to the AI controller. The design of the protocol, where we deliberately overruled the controller, affects naturally the overall performance figures.

### The control group

The swine in the control group were treated by an experienced ICU physician (author MN) according to “best clinical practice”. He attempted to maintain the animal’s blood glucose in the common clinical range of 4.4–9.0 mmol/L based on arterial blood glucose values obtained every 30 min. This scenario was meant to replicate a conventional glucose control treatment scenario whereby an ICU physician at the bedside has access to every 30 min arterial blood glucose values. The broader range in the control group is consistent with the current common glucose range used in Sweden to reduce the risk for hypoglycemia, and in fact would make hypoglycemia less likely than the narrower range used by the AI controller in the treatment group.

The control animals also had their blood glucose continuously measured by an EIRUS system for proper data comparison with the treated group. In the control group, the screen on the EIRUS monitor was covered for the ICU physician. However, he could interfere in both control group and artificial pancreas (AI) group when the EIRUS monitor showed B-glucose values below 2 mmol/L, which was a trigger point for emergency intervention (a “rescue-bolus” of D20 glucose, 20 mL intravenously) per the recommendation of the Animal Ethics Committee. NovoRapid insulin IV injections (1 IU each time) were used for treatment of hyperglycemia, and boluses of D20 glucose (20 mL each time) were given to overcome hypoglycemia. Dosing of insulin and D20 was at the discretion of the ICU physician in order to replicate what occurs in the most ambitious clinical settings without a standardized protocol. The ICU physician was not allowed to use the EIRUS glucose monitor for treatment decisions as the whole point of the experiment was to compare current open loop clinical practice to a fully autonomous closed loop system. In order to avoid comparing intermittent data (Control group) with continuous data (Treated group) we chose to use the continuous glucose data from the EIRUS system in both groups for comparison and statistical analysis.

### The artificial pancreas group

Eight animals were in the artificial pancreas group in which the completely autonomous artificial pancreas system attempted to maintain the animal’s blood glucose in the range of 4.4–6.6 mmol/L. The input to the artificial pancreas system prior to initiating the experiment was the animal’s weight (kg), initial glucose value (mmol/L), desired control range, and concentration of insulin (1 IU/mL) and glucose (D20) used by the artificial pancreas system for purposes of glucose control. The artificial pancreas system received a glucose value from the EIRUS monitor every thirty seconds by a serial asynchronous data communication link. The artificial pancreas system controlled a pair of KD Scientific Legato 100 infusion pumps connected over USB, and that used Becton Dickinson 60 mL syringes for infusion. The artificial pancreas software was run using the LabVIEW 2013 runtime engine on a Lenovo T420 with the latest version of Windows 10. A picture of this setup is available in the supplementary material.

## Data handling and statistical methods

The data were modified when necessary to accommodate for sensor dropout. The first modification was to back-propagate the initial glucose value at a cadence of once per minute to accommodate for tests where the EIRUS sensor had not yet come online at time 0. The second modification was to insert linearly interpolated values between data when sensor values were less frequent than once per minute. When the experiment was ended just prior to time 300 min, as was the case in one control animal, no extra data was appended or extrapolated due to the inherent uncertainty in its validity. Data collected after time 300 min was discarded before analysis.

Once prepared, all data was evaluated for normality using the Shapiro, D’Agostino K^2^, and the Anderson–Darling tests. None of the data passed the normality tests, so the Mann–Whitney *U* test was used for comparison of continuous data, and the Fisher’s exact test for categorical data. A result was considered significant when the p-value was less than 0.05. The glucose data analyzed was the glucose recorded by the EIRUS monitor on an every one-minute interval from time 0 to 300 min in both groups. For each animal, average values were calculated, then from this data a group median ± interquartile range (25–75) was calculated. All statistical calculations were made using the modules available in SciPy 0.18.1 and NumPy 1.11.2, using Python 3.5.2 for Windows (32-bit). The primary safety outcome measurement was the occurrence of severe hypoglycemia (< 2.22 mmol/L). The secondary safety outcome measurements were measures of hypoglycemia including time in range < 2.22 and < 3.5 mmol/L, area under the curve (AUC) 2.22 and 3.5 mmol/L, minimum glucose level, and longest singular hypoglycemic (< 3.5 mmol/L) event. The primary performance measure was “time within target range” for both the controller (4.4–6.6 mmol/L) and the conventional clinical protocol (4.4–9.0 mmol/L).

## Results

**Fig. 2:**
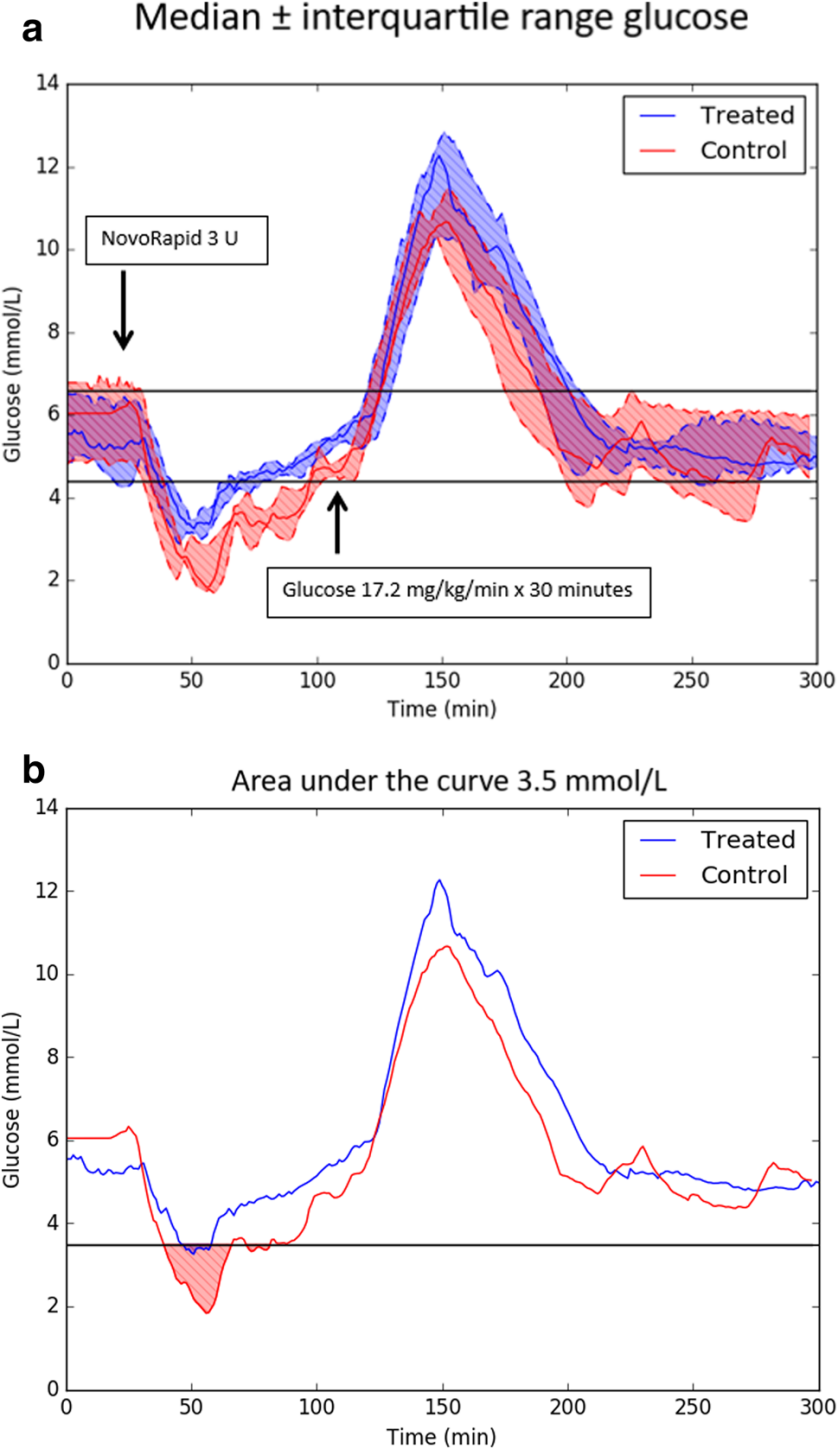
**a** Plot of median ± interquartile range (25–75). Blood glucose trend for the artificial pancreas system (Treated) is presented in blue. The trend for manual control groups (Control) is red. The insulin injection (3 units) given at point 20 min caused a more severe hypoglycemia at time 50 min in Controls compared to Treated. All indices for hypoglycemia were significantly different between groups and in advantage to the subjects controlled by the artificial pancreas system (Treated) (Table 2). **b** For clarity are median glucose values trended separately and without interquartile ranges. To be noticed is that severe hypoglycemia did not occur in the “Treated”-group. The Treated group had a higher peak glucose excursion at time 150 min due to the design of the AI algorithm used in the present study

Table 1 and Fig. 2 present the integrated results from the study.

**Table 1:** Overall glucose control metrics comparing control achieved by autonomous AI-based closed-loop system (Treated) with manual open-loop control attempted by ICU physician (Control). Desired control range was 4.4–6.6 mmol/L for Treated group and 4.4–9.0 mmol/L for Control group. All glucose data were considered in these statistics. “Percent time in range” is presented as median (IQR). *Statistically significant (p < 0.05)

Glucose control metrics	Treated (N = 8)	Control (N = 6)	p value
Percent time in range < 2.22 mmol/L	0.00 (0.00–0.00)	4.14 (1.83–5.52)	< 0.004*
Percent time in range < 3.5 mmol/L	4.16 (3.48–5.32)	13.79 (11.13–20.43)	< 0.004*
Percent time in range 4.4–6.6 mmol/L	55.4 (52.9–59.4)	32.8 (32.4–47.1)	< 0.034*
Percent time in range 4.4–9.0 mmol/L	64.5 (61.3–72.7)	54.5 (43.7–64.3)	0.138
Mean (mmol/L)	6.04 (5.76–6.22)	5.61 (5.09–6.33)	0.438
Standard deviation (mmol/L)	2.23 (1.90–2.57)	2.25 (2.07–2.39)	0.847
Coefficient of variation	0.36 (0.32–0.42)	0.38 (0.36–0.42)	0.477

The artificial pancreas system outperformed the conventional manual treatment executed by an ICU physician regarding glucose control. The total percent time within tight glucose control range, 4.4–6.6 mmol/L, was 55% for Treated and 33% for Controls (p < 0.034). Furthermore, despite the longer time within the tight glucose control range, the artificial pancreas system protected the subjects from both moderate hypoglycemia (B-glucose < 3.5 mmol/L) and severe hypoglycemia (B-glucose < 2.2 mmol/L). Percent time with moderate hypoglycemia and severe hypoglycemia was 4 and 0% for the artificial pancreas system vs 14 and 4% respectively for the Control group (p < 0.004). Table 2 demonstrates that all seven hypoglycemic parameters analyzed were significantly better in the artificial pancreas treated animals, and severe hypoglycemia was abolished in the Treated group (0 Treated vs 5 Control subjects, p < 0.003).

**Table 2:** Hypoglycemia defined to be glucose less than 3.5 mmol/L. All continuous data are reported as median (25–75). Statistical significance (p < 0.05) indicated by *. ^†^If an animal experienced any values less than 2.22 mmol/L, it was counted as having experienced severe hypoglycemia—comparison made using the Fisher’s exact test. ^††^Area under the curve (AUC)—area accumulated while glucose was less than this threshold

Hypoglycemia statistics		Treated	Control	p value
Number of animals with severe hypoglycemia^†^		0	5	< 0.003*
Minimum glucose (mmol/L)		2.83 (2.75–2.96)	1.69 (1.68–1.89)	0.033*
Total time in hypoglycemia for all events (min)		12.5 (10.5–16.0)	41.0 (33.5–61.3)	< 0.004*
Mean of all hypoglycemic glucose values (mmol/L)		3.20 (3.07–3.24)	2.80 (2.43–2.87)	< 0.033*
Longest singular hypoglycemic event (min)		9.5 (8.8–11.3)	24.5 (20.8–29.8)	< 0.012*
AUC^††^ 3.5 mmol/L (mmol/L * min)		4.8 (3.1–5.2)	28.9 (21.1–54.2)	< 0.004*
AUC^††^ 2.2 mmol/L (mmol/L * min)		0.0 (0.0–0.0)	3.6 (1.3–5.2)	< 0.004*

In the subsequent hyperglycemic provocation, with an unphysiological bolus of 20% Dextrose, there was a tendency for the glucose controller to respond slower than the ICU physician. Table 3 demonstrates that in the present study the artificial pancreas system used statistically significantly greater quantities of both insulin (3.7 vs 1 IU, p < 0.002) and glucose (36 vs 12 g, p < 0.008) compared to the manually controlled subjects.

**Table 3:** Summary of insulin/glucose dosing required to maintain desired control range of 4.4–6.6 mmol/L by autonomous AI-based closed-loop system (Treated) and 4.4–9 mmol/L in manual open-loop system (control). The 3 IU of NovoRapid and 15 g of glucose given per the protocol are not included in this analysis. Data are median (25–75). *Statistically significant (p < 0.05)

Interventions dosing metrics	Treated (N = 8)	Control (N = 6)		p value
Total insulin dose given outside of protocol (IU)	3.7 (3.4–4.0)	1 (1.0–1.0)		< 0.002*
Total glucose dose given outside of protocol (grams)	36.3 (27.2–39.8)	12.0 (8.0–16.0)		0.008*

The EIRUS continuous glucose sensor system had an average uptime rate of 95.4 ± 4.1% time in the artificial pancreas group and 96.4 ± 1.9 in the control group. The average EIRUS sensor downtime was 10.5 ± 12.3 min in the artificial pancreas group and 9.5 ± 5.3 min in the control group. There were no significant downtime differences between the groups. The longest EIRUS sensor downtime was 38 min due to failure of the ex-vivo glucose sensor box—occurred in artificial pancreas group. Despite this incident and that the glucose sensor uptime rate was not 100%, the artificial pancreas system had the ability to control blood glucose better than the experienced ICU physician and abolished severe hypoglycemia.

The EIRUS sensor downtime did not affect overall device functionality as the closed loop system was designed to handle periods with no glucose sensor input. The overall device had no downtime during this study. Paired blood gases (N = 115) from the superior vena cava vein the EIRUS glucose sensor catheter resided in resulted in a mean absolute relative difference (MARD) of 15.3% in the artificial pancreas group.

## Discussion

We have in this study demonstrated the safety of an autonomous closed-loop glucose control system comprised of the EIRUS CGM and AI-based glucose control software in a swine model, with unannounced (for the controller) hypoglycemic and hyperglycemic challenges. The important finding from the present study is that an autonomous glucose control system can protect a subject from severe hypoglycemia (< 2.2 mmol/L) even when a very high intravenous insulin dose is given to perturb the system. The autonomous glucose control system also significantly reduced the severity and duration of moderate hypoglycemia (< 3.5 mmol/L) despite attempting to control to a narrower range which should have made it more susceptible to hypoglycemic excursions. This improved safety is important since the major risk for ICU patients with the otherwise beneficial TGC concept is that of moderate and severe hypoglycemia. Our well-monitored control group demonstrates that in clinical practice, even with a broader target range and frequent arterial blood glucose values, it is very difficult to achieve effective glucose control without experiencing significant episodes of hypoglycemia. To this end, and in an effort to minimize hypoglycemia, multiple societies have raised the recommended control range for TGC, which may minimize the benefit observed in studies performed with lower glucose control ranges [19, 29]. However, a wider control range still couldn’t protect the animals in our study from severe hypoglycemia when open loop control was used (Control group).

Use of a closed-loop glucose control system that has access to frequent glucose data (e.g., every 30 s) will be the only way to achieve effective TGC to a lower range while simultaneously minimizing hypoglycemic episodes. A closed loop system will require accurate glucose data at a minimum of every 5 min and must be able to adjust the infusion rates of insulin and/or glucose every 5–10 min, to achieve safe and effective glucose control. Open-loop systems that adjust infusion rates every 1–4 h and rely on human intervention will never be able to provide completely safe (e.g., no hypoglycemia) glucose control.

The artificial pancreas system demonstrated its ability to abolish severe hypoglycemia and minimize moderate hypoglycemia, while at the same time improving upon the time in the tight glucose range of 4.4–6.6 mmol/L. Our high glucose infusion rate was meant to overrule the controller in order to create hyperglycemia (> 10 mmol/L) and assess for the occurrence of “rebound” hypoglycemia secondary to the insulin doses used to treat the hyperglycemic excursion. We did note the occurrence of a higher peak in the treatment group’s glucose value during the glucose infusion portion of the experiment, and feel there is room for improvement in the controller’s algorithms responsible for reducing its glucose output during periods of hyperglycemia. This study was of too short duration (e.g., 5 h) and the planned glucose excursions too extreme to have given the AI controller a chance to maintain the glucose level in the desired range, which explains its inability to achieve improved performance with regards to the glucose variability metrics. However, in our comparative simulation study, our AI-based controller’s variability (CV) was 46 percent lower than the next best controllers [26].

The artificial pancreas system functioned in an autonomous mode and overall required less work to operate, which is an important feature considering nurses will spend up to 2 h/patient/day to achieve TGC [30]. If the systems glucose infusion is delivered via a standard infusion pump with a one-liter reservoir bag, the artificial pancreas system will require no work to operate other than that required at initiation of the system – typically less than 5 min.

The artificial pancreas system did use more insulin and glucose to achieve its improved performance. This is not unexpected as any real-time biomedical control system that infuses regulatory (insulin) and counter-regulatory (glucose) substances is likely to use more of each substance as it attempts to maintain control of a highly variable system and is consistent with the results of our comparative simulation study [26].

To our knowledge, this study represents the first time a CE-marked CGM that was designed for use in the ICU setting has been integrated with glucose control software designed for use in the ICU setting to create a fully autonomous glucose control system. An advantage the EIRUS CGM has over other CE-marked CGM systems [31, 32] is that its microdialysis sensing method does not require aspiration of blood across an ex-vivo glucose sensor. Systems that require aspiration of blood will be more prone to failure from inability to aspirate blood from the catheter due to biofilm, thrombus, or position of the catheter against the venous/arterial wall [33–37]. Although the EIRUS CGM demonstrated a MARD of 15.33% in the treated group, a recent clinical study done over a longer period in cardiac surgery patients demonstrated a MARD of 6.5% across 514 paired samples [23].

The EIRUS CGM system had an average uptime rate of more than 95% in both groups. The interruptions that were seen were the result of small gas bubbles passing by the glucose sensor. When the sensor detects a bubble, an automatic flush of the microdialysis channel is started. The brief sensor down time seen in this study did not affect the overall performance of the artificial pancreas system.

The AI-based glucose control software was patterned after the native system of man [24]. One advantage this controller has is that it was designed to operate in a base cycle frequency of every 10 min but will automatically change to a cycle frequency of every 5 min during states of hypoglycemia or extreme glucose fluctuation. This feature gives it stability during states of low glucose rate of change (ex <  ± 0.056 mmol/L/min), but also allows it to quickly adapt when either the rate of change is large (ex ≥  ± 0.056 mmol/L/min) or the patient is experiencing relative/absolute hypoglycemia.

The two main components of the artificial pancreas system—EIRUS CGM and the AI-based glucose control software—integrated well together and operated in a nominal fashion with no errors in system integration.

## Limitations of study

This safety and performance study was limited by use of an experimental protocol that was not an adequate substitute for the stress induced hyperglycemia experienced by ICU patients, and in addition used overdoses of both insulin and glucose that have no clinical correlate.

## Future steps

In order to bring this artificial pancreas system to the clinic as an approved product the following challenges will need to be overcome: (1) performing initial smaller clinical tests of the AI-based glucose control software to establish its safety and efficacy in a real clinical environment (e.g., cardiac ICU, medical ICU), (2) incorporation of the EIRUS CGM system into an integrated device with user interface and intravenous pumps for insulin and dextrose infusion, (3) performing a pivotal multi-center study of the final integrated device under the coordination of a contract research organization, with this study being led by a U.S. academic research team with inpatient glucose control study experience.

## Conclusion

In this swine study, the combination of an accurate continuous blood glucose monitoring system with AI-based glucose control software abolished all severe hypoglycemic events despite a grave hypoglycemic provocation. The artificial pancreas system also demonstrated its ability to minimize moderate hypoglycemia excursions despite having to deal with an unannounced intravenous insulin injection.

## Data Availability

The datasets used and/or analyzed during the current study are available from the corresponding author on reasonable request.

## Abbreviations

AI: Artificial intelligence
AUC: Area under the curve
B: Blood
B-glucose: Blood glucose
CE: European conformity
CGM: Continuous glucose monitor
cm: Centimeters
CVC: Central venous catheter
FiO_2_: Fraction of inspired oxygen
H_2_O: Water
hr: Hour
ICU: Intensive care unit
IM: Intramuscularly
IMT: Ideal medical technologies
IU: International unit
IV: Intravenously
kg: Kilograms
MARD: Mean absolute relative difference
mcg: Microgram
mg: Milligrams
min: Minute
ml: Milliliter
mmol/L: Millimole/Liter
MPC: Model predictive control
N: Number
PD: Proportional derivative
PEEP: Positive end expiratory pressure
TGC: Tight glucose control
USB: Universal serial bus
